# Comparative effectiveness of dopamine agonists and monoamine oxidase type-B inhibitors for Parkinson’s disease: a multiple treatment comparison meta-analysis

**DOI:** 10.1007/s00228-020-02961-6

**Published:** 2020-07-24

**Authors:** Caroline D. Binde, Ingunn F. Tvete, Jørund I. Gåsemyr, Bent Natvig, Marianne Klemp

**Affiliations:** 1 Department of Pharmacology, University of Oslo, Oslo, Norway; 2 Norwegian Computing Centre, Oslo, Norway; 3 Department of Mathematics, University of Oslo, Oslo, Norway

**Keywords:** Dopamine agonists, MAO-B inhibitors, Multiple treatment comparison, Parkinson’s disease, Effectiveness, Serious adverse events

## Abstract

**Purpose:**

To investigate the comparative effectiveness of dopamine agonists and monoamine oxidase type-B (MAO-B) inhibitors available for treatment of Parkinson’s disease.

**Methods:**

We performed a systematic literature search identifying randomized controlled trials investigating 4 dopamine agonists (cabergoline, pramipexole, ropinirole, rotigotine) and 3 MAO-B inhibitors (selegiline, rasagiline, safinamide) for Parkinson’s disease. We extracted and pooled data from included clinical trials in a joint model allowing both direct and indirect comparison of the seven drugs. We considered dopamine agonists and MAO-B inhibitors given as monotherapy or in combination with levodopa. Selected endpoints were change in the Unified Parkinson’s Disease Rating Scale (UPDRS) score, serious adverse events and withdrawals. We estimated the relative effectiveness of each dopamine agonist and MAO-B inhibitor versus comparator drug.

**Results:**

Altogether, 79 publications were included in the analysis. We found all the investigated drugs to be effective compared with placebo when given as monotherapy except safinamide. When considering combination treatment, the estimated relative effects of selegiline, pramipexole, ropinirole, rotigotine, cabergoline, rasagiline and safinamide were 2.316 (1.819, 2.951), 2.091 (1.889, 2.317), 2.037 (1.804, 2.294), 1.912 (1.716, 2.129), 1.664 (1.113, 2.418), 1.584 (1.379, 1.820) and 1.179 (1.031, 1.352), respectively, compared with joint placebo and levodopa treatment.

**Conclusions:**

Dopamine agonists were found to be effective as treatment for Parkinson’s disease, both when given as monotherapy and in combination with levodopa. Selegiline and rasagiline were also found to be effective for treating Parkinson’s disease, and selegiline was the best option in combination with levodopa among all the drugs investigated.

**Electronic supplementary material:**

The online version of this article (10.1007/s00228-020-02961-6) contains supplementary material, which is available to authorized users.

## Introduction

Pharmacological treatment of Parkinson’s disease is complex, as there are several treatment options available, but little information on how these options compare. The main therapeutic strategy for Parkinson’s disease has been replacement of dopamine, via the dopamine precursor levodopa [1, 2]. However, chronic treatment with levodopa is complicated by the development of motor fluctuations, wearing-off effect and random switches between “on” and “off” states [2]. Up to 40% of patients treated with levodopa for 5 years or more will experience end-of-dose deterioration [3].

There are several agents available for the treatment of Parkinson’s disease, and both dopamine agonists and monoamine-oxidase type B (MAO-B) inhibitors can be used alone or in combination with each other or with levodopa. When starting treatment, it is in the best interest of the patient to identify the most effective and safe option from a range of alternatives, as well as to consider whether it is most important to obtain control over motor symptoms or to delay development of levodopa side effects. For younger patients, it would be desirable if an alternative treatment option to levodopa could delay the need for levodopa and hence the side effects associated with chronic levodopa treatment. Both dopamine agonists and MAO-B inhibitors are available as alternatives to levodopa, but there is no clear evidence that one of these options is better than the other. Therefore, the comparative effectiveness of dopamine agonists and MAO-B inhibitors, both when given alone and in combination with levodopa, needs to be better established.

We have previously investigated the comparative effectiveness of MAO-B inhibitors available for treatment of Parkinson’s disease [4]. We conducted a multiple treatment comparison (MTC) meta-analysis assessing which drug had the highest probability of being the most effective drug for early and late Parkinson’s disease. We evaluated both clinical improvement and serious adverse events (SAE). We found that all of the included MAO-B inhibitors (selegiline, rasagiline and safinamide) were effective compared to placebo, both when given alone and in combination with levodopa. When considering combination therapy with MAO-B inhibitors and levodopa, we found that selegiline was the most effective drug [4].

Other reviews have previously compared several drugs used for treatment of Parkinson’s disease, but we could not identify any studies performing a comprehensive comparison with dopamine agonists and MAO-B inhibitors available for treatment of Parkinson’s disease, both when used as monotherapy and in addition to levodopa. We did a systematic MEDLINE search for systematic reviews and meta-analyses comparing pharmacological treatment for Parkinson’s disease, and we found only a few publications. One Cochrane review investigated three drug classes assessing the benefits and risks of these drugs when used in the treatment of patients suffering from Parkinson’s disease with motor complications [5]. This review compared catechol-O-methyl transferase (COMT) inhibitors, MAO-B inhibitors and dopamine agonists with placebo when used in combination with levodopa. They found that treatment with dopamine agonists may be more effective than treatment with MAO-B inhibitors and COMT inhibitors in managing symptoms of Parkinson’s disease, but regarding dopamine agonists and MAO-B inhibitors, they found no significant differences between individual drugs within each drug class [5].

Li et al. conducted a network meta-analysis comparing ten drugs used in the treatment of non-motor symptoms of Parkinson’s disease [6]. They included trials involving drugs from different drug classes (ropinirole, rasagiline, rotigotine, entacapone, apomorphine, pramipexole, sumarinole, bromocriptine, piribedil and levodopa). They found that among the drugs included in their analysis, apomorphine appeared to be the most efficacious [6].

Zhuo et al. did a comprehensive comparison of ten drugs used in the treatment of Parkinson’s disease [7]. Their study was designed to investigate efficacy and tolerability of ten drugs used as monotherapy in the treatment of Parkinson’s disease. They found that levodopa, selegiline, ropinirole and rotigotine showed effectiveness and could be recommended as treatment for patients with Parkinson’s disease [7]. We think it is important to also investigate the comparative effectiveness of these agents when given in combination with levodopa, and we have therefore included studies examining this. Levodopa is almost unavoidably added to the treatment of Parkinson’s disease after a few years, to keep control of the progressive symptoms [2, 8].

We therefore extended our previous research [4] to investigate the comparative effectiveness of both dopamine agonists and MAO-B inhibitors available for treatment of Parkinson’s disease. We performed a comprehensive literature search and pooled data from all relevant published clinical trials involving four dopamine agonists (cabergoline, pramipexole, rotigotine and ropinirole). We also included published clinical trials considering MAO-B inhibitors from our previous publication [4], allowing both direct and indirect comparisons of all seven drugs in a joint model. There is no single clinical trial actively comparing all dopamine agonists and MAO-B inhibitors, but we can pool data from published clinical trials in an MTC analysis simultaneously to assess which drug has the highest probability of being the most effective or the safest option, both when given alone and in combination with levodopa. Additionally, disease duration, dose level and duration of study could influence the effect and SAE of the various treatments and the degree of withdrawal from the study. We explored this in our analysis.

## Methods

### Literature search

**Fig. 1:**
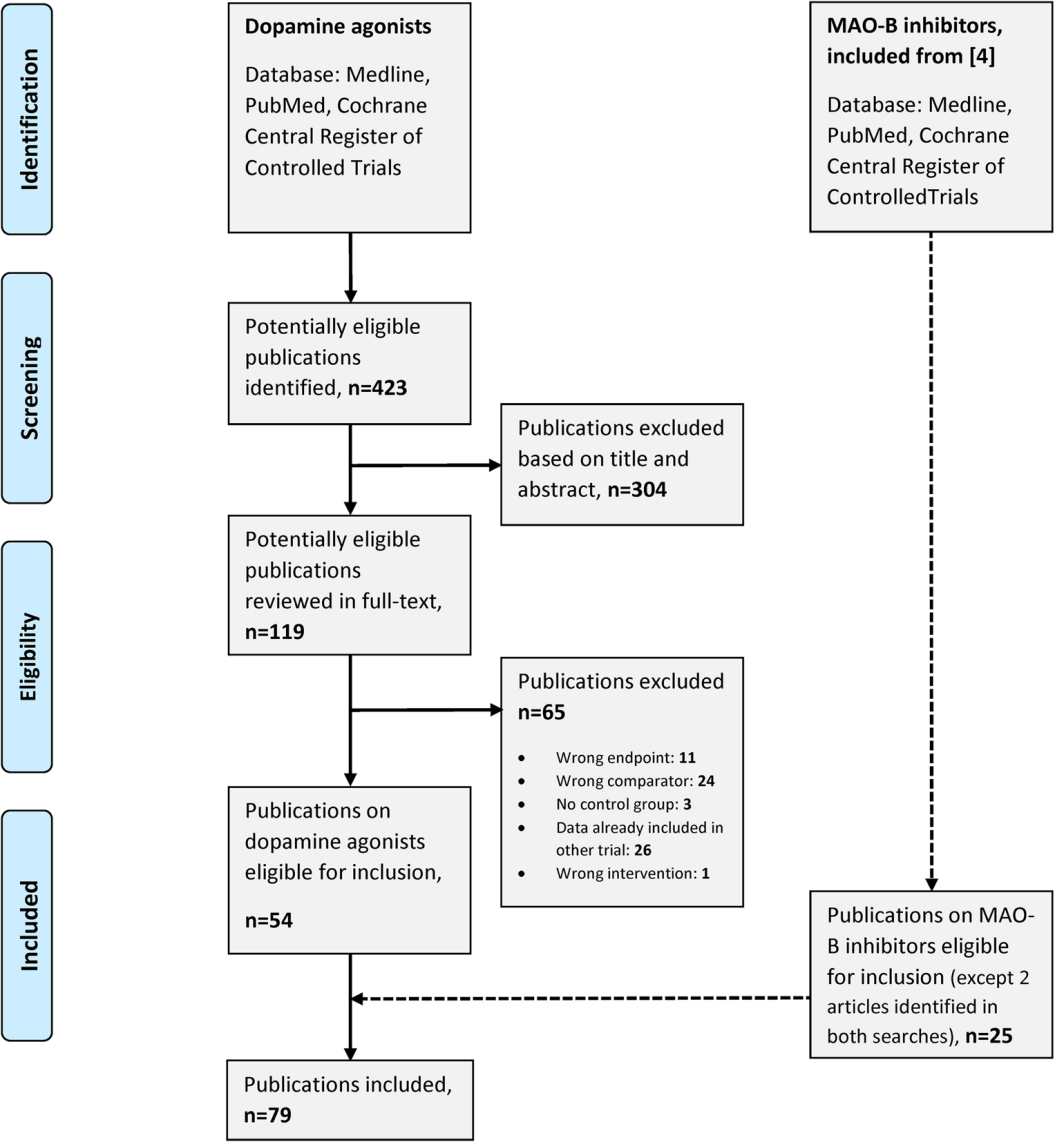
Identification and selection of publications. MAO-B inhibitors included and adapted from [4]

We performed a systematic literature search, using MEDLINE, PubMed and Cochrane Central Register of Controlled Trials, to identify randomized controlled trials (RCTs) assessing the efficacy of dopamine agonists in patients with Parkinson’s disease. We included dopamine agonists (cabergoline, pramipexole, apomorphine, ropinirole and rotigotine) and indication (Parkinson’s disease) as search terms and limited our search to RCTs (Appendix S1). Two researchers screened the list of potentially eligible clinical trials by title and/or abstract. We retrieved potentially eligible publications for full-text review to determine whether they met our pre-specified inclusion criteria. Publications that included men and women with Parkinson’s disease aged 18 years or older, comparing the interventions of interest (cabergoline, pramipexole, ropinirole or rotigotine) with each other or placebo, with or without additional levodopa, were eligible. We found the clinical trials assessing the effectiveness of apomorphine to differ too much in administration form (infusion, injection, inhalation and sublingual administration). We therefore decided to exclude studies on apomorphine from the analysis. We searched through reference lists to identify additional trials. The search was conducted on 28 September 2017. We also included 25 publications from our previous review assessing the efficacy of MAO-B inhibitors [4]. Both searches were last updated in May 2019. Details of the identification and selection of publications are displayed in the PRISMA flowchart (Fig. 1).

### Participants and study selection

Two researchers independently reviewed the full-text publications and extracted data from the publications that met our pre-specified inclusion criteria. We included publications presenting RCTs of patients with Parkinson’s disease above the age of 18, evaluating the efficacy or safety of dopamine agonists (cabergoline, pramipexole, ropinirole or rotigotine) or MAO-B inhibitors (selegiline, rasagiline or safinamide), given either as monotherapy or in combination with levodopa. According to our study protocol (Appendix S2) which was defined a priori, we extracted data on outcomes of interest, which were change in Unified Parkinson’s Disease Rating Scale (UPDRS) [9] score (responders), serious adverse events, withdrawals (discontinuation of drug use), mortality and need for levodopa. There were very few deaths, and we did not have resources to investigate the need for levodopa in depth. Therefore, we present the results regarding the number of responders, serious adverse events and withdrawals in this paper. Publications were excluded if they failed to meet our inclusion criteria regarding trial design, patient population, intervention, comparator or endpoints. Risk of bias of included studies is assessed at study level using the risk of bias tool described in Cochrane Handbook for Systematic Reviews of Interventions and is available in the supplementary materials (Appendix S3). The Cochrane risk-of-bias tool for randomized trials (RoB2) [10] was used to assess the risk of bias across five domains: the randomization process, assignment to intervention, missing outcome data, measurement of the outcome and selection of the reported result. Studies showing high risk of bias in two or more domains were excluded from the analysis.

Responders were defined as the number of patients with an improvement (minimally improved, much improved or very much improved) on the Clinical Global Impressions (CGI) scale [11] or with at least 20% reduction in the UPDRS score from baseline to end of study. The UPDRS total score was used where it was provided; the activities of daily living (ADL) sub-score (part II) and/or the motor sub-score (part III) were used where only these were provided. Entacapone, a catechol-O-methyltransferase (COMT) inhibitor, was used in combination with levodopa as a comparator in one of the included clinical trials, and was therefore indirectly included in the analysis, but was not a drug we focused on.

### Data

**Fig. 2:**
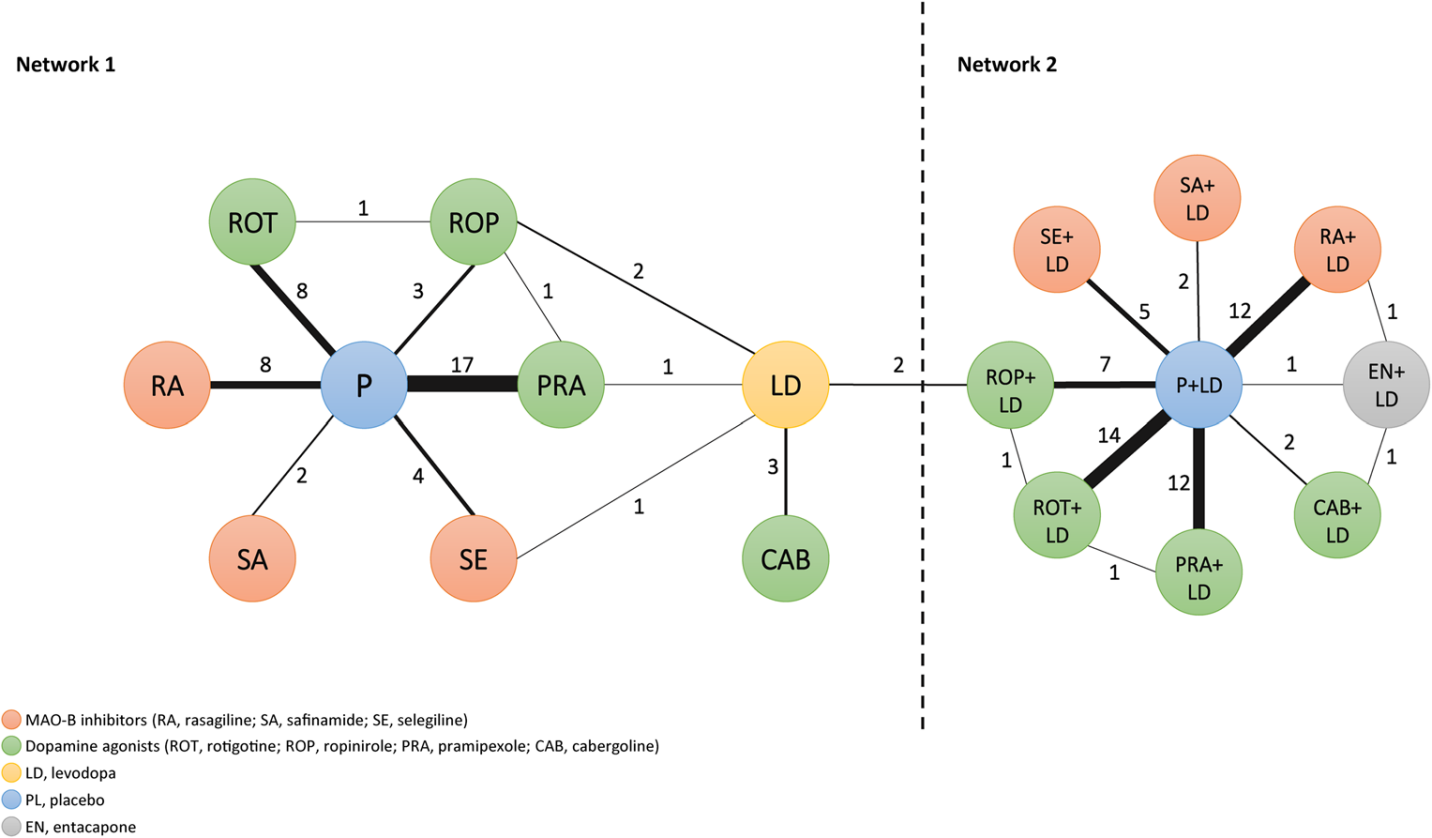
Overview of direct and indirect comparisons. The numbers and the thickness of the lines indicate the number of clinical trials in each comparison

We originally found two studies comparing levodopa and ropinirole to levodopa, giving one complete network embracing all treatments from the 79 studies. However, when considering this network, we ran into inconsistency issues. We therefore decided to analyse two separate networks, one considering monotherapy treatments with placebo as the comparator treatment and another considering combination treatment with dopamine agonists or MAO-B inhibitors and levodopa with placebo and levodopa treatment as the comparator treatment. We will refer to these two networks as, respectively, networks 1 and 2 (Fig. 2).

We defined disease duration as short (less than 3 years) or long (3 years or more). Dose level was defined as low or high with individual cut-off levels for the different Parkinson drugs (Appendix S4). Duration of study was defined as short (less than 26 weeks) or long (26 weeks or more).

### Statistical analysis

For both networks, we constructed a joint model for assessing the comparable relative effects, the relative risk of withdrawal and the relative risk of serious adverse events between the treatments for each treatment versus the relevant comparator, following Tvete et al. [12]. The relevant comparators were placebo and joint placebo and levodopa treatment in the first and second network, respectively. All treatment arms over all studies in a network contributed to the comparison of all drugs relative to each other. We give a detailed presentation of the model in the supplementary material (Appendix S5).

In our Bayesian modelling approach, we estimated, taking into consideration the study data, the posterior distribution of the relative effect of one drug versus another. We addressed the possible presence of heterogeneity by adjusting for known relevant factors as suggested in Higgins et al. 2003 [13]. We hence considered models where we related the treatments’ effect to the disease duration, dose level and study duration, giving a regression coefficient in each case to be estimated.

We analysed the models in OpenBUGS [14] run from R [15]. In network 1, we sampled from the posterior distribution of the relative effect of each drug versus each other and versus placebo. In network 2, we sampled for the posterior distribution of the relative effect of each drug in combined treatment with levodopa versus each other in combined treatment with levodopa and versus joint placebo and levodopa treatment. Similarly, in models adjusting for either dose level, duration of disease or duration of study, we sampled from the posterior distribution of the respective regression coefficients. Based on the posterior samples, we estimated all parameters entering the model, including the relative effects. A visual inspection of the MCMC chains and computing Rhat [16], the potential scale reduction factor, for the parameters entering the models were done to check for convergence issues.

We present all estimates with corresponding 95% uncertainty (credibility) intervals. Based on the posterior samples, we could estimate the probability that one treatment was better than another by counting the number of times; the corresponding relative effect was greater. Similarly, we could estimate the probability that a treatment was ranked as number 1 and 2.

## Results

We identified 423 potentially eligible publications assessing the efficacy of dopamine agonists, where 304 were excluded based on title and abstract. One hundred and nineteen publications were retrieved for full-text review. Of these, 65 were found to be not relevant and were excluded (Appendix S6). Fifty-four publications on dopamine agonists and 25 publications on MAO-B inhibitors from our previous review [4] were included. Altogether, 79 publications were included in the multiple treatment comparison analysis (Appendix S7) [17–95].

The 79 publications included a total of 20,773 patients, of which 8381 received treatment with a dopamine agonist (given as monotherapy or in combination with levodopa) and 3736 received a MAO-B inhibitor (given as monotherapy or in combination with levodopa). A total of 3386 patients received placebo and 4077 received placebo and levodopa. Eight hundred and eighty-four patients received levodopa only, and 309 patients received entacapone. The average disease duration ranged from 3 months to almost 14 years. A total of 9036 patients had disease duration of less than 3 years, and 11,737 patients had disease duration of 3 years or more. The durations of the clinical trials ranged from 6 weeks to six and a half years, most of them lasting between 12 and 36 weeks.

The number of responders and serious adverse events extracted from the studies are presented in the supplementary materials (Appendix S8 and S9). Figure 2 displays the two networks of direct and indirect comparisons. Altogether, there are 51 comparisons in network 1 (monotherapy) and 59 in network 2 (combination therapy) (Fig. 2 and Appendix S7). All of the included clinical trials are considered to have low or medium risk of bias (Appendix S3).

### Treatment effect

#### Network 1

**Fig. 3:**
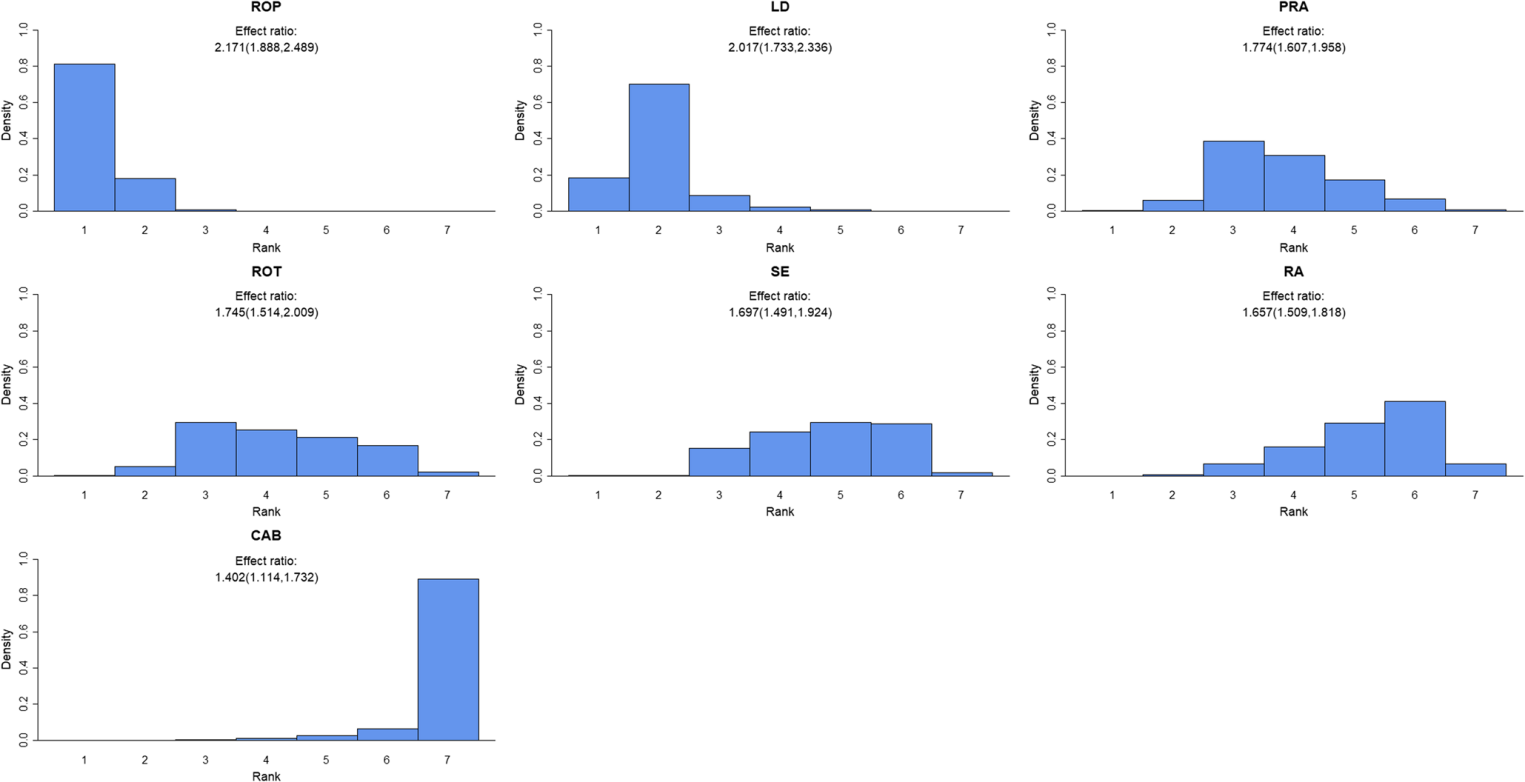
Histograms displaying a given dopamine agonist or MAO-B inhibitor’s effect ranked against the other drugs (ranked from left to right) when given as monotherapy. The height of the bars gives the probability of being ranked as number one to seven. The effect ratios are the estimated effect of given drug versus placebo treatment. ROP, ropinirole; LD, levodopa; PRA, pramipexole; ROT, rotigotine; SE, selegiline; RA, rasagiline; CAB, cabergoline

Analysing network 1 without taking dose level, duration of disease or duration of study into account, we found monotherapy with dopamine agonists (cabergoline, pramipexole, rotigotine and ropinirole), MAO-B inhibitors (selegiline, rasagiline and safinamide) and levodopa, to be effective compared with placebo, except safinamide. We found ropinirole to be the most effective option, followed by levodopa. Next pramipexole, rotigotine, selegiline and rasagiline were of similar effect, followed by cabergoline. The estimated relative effects are 2.171 (1.888, 2.489), 2.017 (1.733, 2.336), 1.774 (1.607, 1.958), 1.745 (1.514, 2.009), 1.697 (1.491, 1.924), 1.657 (1.509, 1.818) and 1.402 (1.114, 1.732) respectively (Table 1). The effect estimate for safinamide was similar to that of cabergoline but was associated with large uncertainty, the credibility interval containing 1. Figure 3 displays the ranking of the dopamine agonists and the MAO-B inhibitors when given alone. The probability that one drug is better than another is displayed in Table 2. We found 82% probability for ropinirole to be better than levodopa and a 99% probability for ropinirole to be better than pramipexole. Similarly, there is a 93% probability for levodopa to be better than pramipexole (Table 2).

**Table 1:** UPDRS responders, serious adverse events and withdrawals in the networks; effect ratio estimates

		MAO-B inhibitors			Dopamine agonists				Other
		RA	SA	SE	CAB	PRA	ROP	ROT	LD
Network 1	UPDRS responders^a^	1.657 (1.509, 1.818)	1.468 (0.888, 2.393)	1.697 (1.491, 1.924)	1.402 (1.114, 1.732)	1.774 (1.607, 1.958)	2.171 (1.888, 2.489)	1.745 (1.514, 2.009)	2.017 (1.733, 2.336)
	UPDRS responders^b^	1.797 (1.675, 1.926)	1.361 (0.836, 2.074)	1.663 (1.463, 1.884)	1.329 (1.063, 1.642)	1.763 (1.614, 1.919)	1.953 (1.647, 2.266)	1.552 (1.373, 1.749)	1.915 (1.638, 2.232)
	Serious adverse events	1.048 (0.613, 1.709)	1.054 (0.325, 2.461)	0.789 (0.285, 1.714)	1.026 (0.567, 1.664)	2.021 (1.394, 2.885)	1.163 (0.765, 1.645)	0.900 (0.624, 1.245)	0.833 (0.482, 1.313)
	Withdrawals	0.865 (0.648, 1.089)	0.954 (0.601, 1.361)	1.175 (0.86, 1.592)	0.985 (0.765, 1.229)	1.104 (0.926, 1.283)	0.848 (0.728, 0.979)	1.091 (0.922, 1.293)	0.785 (0.628, 0.951)
		RA + LD	SA + LD	SE + LD	CAB + LD	PRA + LD	ROP + LD	ROT + LD	EN + LD
Network 2	UPDRS responders^a^	1.584 (1.379, 1.82)	1.179 (1.031, 1.352)	2.316 (1.819, 2.951)	1.664 (1.113, 2.418)	2.091 (1.889, 2.317)	2.037 (1.804, 2.294)	1.912 (1.716, 2.129)	1.429 (1.16, 1.74)
	UPDRS responders^b^	1.544 (1.349, 1.762)	1.217 (1.066, 1.392)	2.503 (1.946, 3.222)	1.455 (1.006, 2.068)	2.093 (1.891, 2.316)	2.095 (1.861, 2.356)	1.933 (1.737, 2.149)	1.312 (1.098, 1.570)
	Serious adverse events	1.052 (0.812, 1.405)	1.043 (0.837, 1.343)	1.045 (0.818, 1.394)	0.969 (0.652, 1.281)	1.034 (0.806, 1.337)	1.012 (0.799, 1.278)	1.030 (0.791, 1.352)	1.006 (0.755, 1.323)
	Withdrawals	0.903 (0.690, 1.201)	1.113 (0.782, 1.571)	0.955 (0.774, 1.159)	0.854 (0.522, 1.334)	0.616 (0.524, 0.72)	0.615 (0.526, 0.713)	0.809 (0.690, 0.945)	0.957 (0.654, 1.34)

^a^Model without taking dose level, duration of disease or duration of study into consideration

^b^Model taking duration of study into consideration

*RA*, rasagiline; *SA*, safinamide; *SE*, selegiline; *CAB*, cabergoline; *PRA*, pramipexole; *ROP*, ropinirole; *ROT*, rotigotine; *LD*, levodopa; *EN*, entacapone

**Table 2:** Probabilities that one drug is better than another regarding responders, in a model without dose level, duration of disease or duration of study

Probability that one drug is better than another given alone							
	LD	PRA	ROT	SE	RA	CAB	SA
ROP	0.82	0.99	1	1	1	1	0.94
LD	-	0.93	0.94	1	0.99	1	0.90
PRA	-	-	0.58	0.72	0.84	0.98	0.81
ROT	-	-	-	0.63	0.72	0.96	0.79
SE	-	-	-	-	0.61	0.97	0.76
RA	-	-	-	-	-	0.92	0.73
CAB	-	-	-	-	-	-	0.48
Probability that one drug is better than another in combination with levodopa							
	PRA + LD	ROP + LD	ROT + LD	CAB + LD	RA + LD	EN + LD	SA + LD
SE + LD	0.76	0.81	0.92	0.93	1	1	1
PRA + LD	-	0.64	0.94	0.89	1	1	1
ROP + LD	-	-	0.83	0.86	1	1	1
ROT + LD	-	-	-	0.78	0.98	0.99	1
CAB + LD	-	-	-	-	0.56	0.76	0.94
RA + LD	-	-	-	-	-	0.87	1
EN + LD	-	-	-	-	-	-	0.94

*RA*, rasagiline; *SA*, safinamide; *SE*, selegiline; *CAB*, cabergoline; *PRA*, pramipexole; *ROP*, ropinirole; *ROT*, rotigotine; *LD*, levodopa; *EN*, entacapone

We found no significant difference in treatment effect for patients with high-dose compared with low-dose level or for patients with short compared with long disease duration, i.e. the coefficients for dose level and disease duration were not significantly different from zero. However, the coefficient for duration of study was significantly different from 0. Hence, the model including an effect of study duration was the model best supported by the data.

Taking duration of study into consideration, we found an increased effect with longer duration of study. After adjusting for duration of study, rasagiline receives a better ranking and is ranked as number three together with pramipexole, following ropinirole and levodopa (Table 1). There were short duration of study in 44 treatment arms and long duration of study in 41 treatment arms.

#### Network 2

**Fig. 4:**
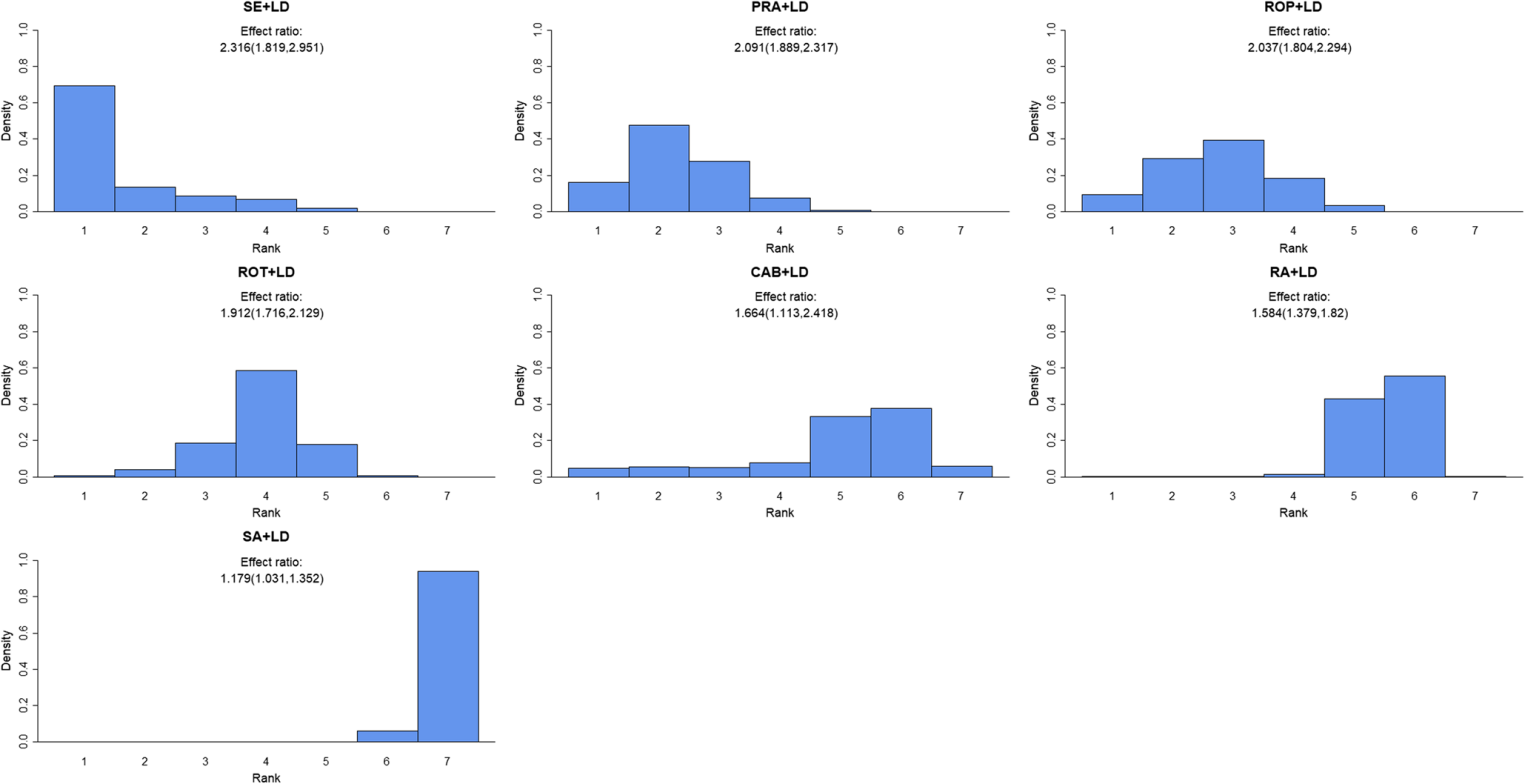
Histograms displaying given dopamine agonist or MAO-B inhibitor’s effect ranked against the other drugs (ranked from left to right) when given in combination with levodopa. The height of the bars gives the probability of being ranked as number one to seven. The effect rations are the estimated effect of the given drug versus placebo treatment when given in combination with levodopa. SE, selegiline; PRA, pramipexole; ROP, ropinirole; ROT, rotigotine; CAB, cabergoline; RA, rasagiline; SA, safinamide

Regarding treatment with a dopamine agonist or a MAO-B inhibitor in combination with levodopa, we found all of the included drugs to be effective compared with placebo. We found selegiline to be the most effective option, followed by pramipexole and ropinirole, rotigotine, cabergoline and rasagiline, and safinamide. The estimated relative effects are 2.316 (1.819, 2.951), 2.091 (1.889, 2.317), 2.037 (1.804, 2.294), 1.912 (1.716, 2.129), 1.664 (1.113, 2.418), 1.584 (1.379, 1.820) and 1.179 (1.031, 1.352) respectively (Table 1). The ranking of the drugs when given in combination with levodopa is displayed in Fig. 4. Table 2 displays the probability that one agent is better than another. We found a 76% probability for selegiline to be better than pramipexole and 81% probability for selegiline to be better than ropinirole when given as combination therapy. Similarly, we find a 64% probability for pramipexole to be better than ropinirole when given together with levodopa (Table 2).

Taking the dose level or disease duration into consideration, we found an increased effect with a high-dose level compared with a low-dose level and similarly an increased effect for those with long disease duration compared with having short disease duration. For both cases, the ranking of the drugs did not change. Considering duration of the study, the coefficient for study duration was close to but just above zero (lower level in the uncertainty interval was less than 0.00003). The ranking of the drugs is the same as without considering study duration, with the exception that rasagiline and cabergoline switched rank, with the estimated relative effects 1.544 (1.349, 1.762) and 1.455 (1.006, 2.068), respectively (Table 1). Hence, rasagiline and cabergoline remain similar in effect when taking study duration into account.

### Serious adverse events

In network 1, we find an increased risk of serious adverse events for treatment with pramipexole compared with placebo (Table 1). For network 2, we find no increased risk of serious adverse events for any of the drugs compared with placebo (Table 1). There were altogether few serious adverse events, and we did not consider patients’ dose level, disease duration or the study duration for the serious adverse event endpoint.

### Withdrawals

Considering withdrawals in network 1, we found no increased risk of withdrawals for any of the drugs compared with placebo. However, we find a significantly lower risk of withdrawals for treatment with ropinirole and levodopa compared with placebo, 0.848 (0.728, 0.979) and 0.785 (0.628, 0.951), respectively (Table 1). In network 2 (combination therapy), we find no increased risk of withdrawals for any of the drugs compared with placebo, but we found a significantly lower risk of withdrawals for treatment with pramipexole, ropinirole and rotigotine, 0.616 (0.524, 0.720), 0.615 (0.526, 0.713) and 0.809 (0.690, 0.945), respectively (Table 1). There were altogether relatively few withdrawals, and we did not consider patients’ dose level, disease duration or the study duration for the withdrawal endpoint.

## Discussion

There is a variety of medical interventions available for the symptomatic treatment of Parkinson’s disease, but there is little information on how these options compare. We aimed to do a comprehensive comparison of dopamine agonists and MAO-B inhibitors available for treatment of Parkinson’s disease, both when given alone and in combination with levodopa.

We included 79 clinical trials including a total of 20,773 patients. Our results suggest that both dopamine agonists and MAO-B inhibitors are effective as monotherapy treatment for patients with Parkinson’s disease. We found the dopamine agonist ropinirole to be the best treatment. Noticeably, we found ropinirole to be ranked higher than levodopa when given as monotherapy. However, we did not actively search for clinical trials comparing levodopa with placebo, so we cannot exclude the possibility that we are lacking evidence on this part. We found a considerable variation in treatment effect within each drug class, especially within the class of dopamine agonists.

When considering combination treatment for Parkinson’s disease, we found selegiline to be the most effective drug in combination with levodopa. These results are in line with the results of our previous publication, where we investigated the efficacy and safety of three MAO-B inhibitors (selegiline, rasagiline, and safinamide) and found selegiline to be the most effective option when given in combination with levodopa [4]. Interestingly, selegiline remains the most effective drug in combination with levodopa after adding all the evidence connected to four dopamine agonists to the analysis. Also for combination treatment, we found considerable variation within each drug class, especially for MAO-B inhibitors. Except for selegiline, no other MAO-B inhibitor was ranked higher than a dopamine agonist when used in combination with levodopa.

It has previously been reported that MAO-B inhibitors appear to have weaker anti-Parkinsonian effect than levodopa [96, 97] and dopamine agonists [97]. Our results support these findings only to some extent. Regarding monotherapy, we found that MAO-B inhibitors appear less effective than the dopamine agonist ropinirole and levodopa. We found selegiline and rasagiline to be the best of the three MAO-B inhibitors included in the analysis for monotherapy. However, it has also been reported a beneficial association between the duration of treatment with MAO-B inhibitors and the degree of clinical worsening [98]. The durations of the clinical trials in our analysis ranged from 6 weeks to six and a half years, most of them lasting between 12 and 36 weeks. When adjusting for duration of study, we found in general an increased effect with longer duration of study. Interestingly, after adjusting for the duration of study, rasagiline received a better ranking and was ranked as number three following ropinirole and levodopa.

Dopamine agonists are associated with more side effects [97, 99], and we found an increased risk of serious adverse events for patients treated with pramipexole. However, we found no increased risk of withdrawals from any of the drugs used as monotherapy compared with placebo. In fact, we found a significantly lower risk of withdrawal for treatment with ropinirole and levodopa, compared with placebo. Regarding combination therapy, we found no increased risk of withdrawals compared with placebo. On the contrary, we found a reduced risk of withdrawals in the groups treated with pramipexole, ropinirole and rotigotine in combination with levodopa, compared with placebo in combination with levodopa. This could suggest that patients tolerate the treatment well, even though there might be side effects, or that the experience of improved health effects outweighs the experience of possible side effects.

Comparisons of different treatment options for Parkinson’s disease have previously been reported, although we could not identify any review comparing all dopamine agonists and MAO-B inhibitors available for treatment of Parkinson’s disease, both when used as monotherapy and in combination with levodopa. Zhuo et al. [7] recommend, in a comprehensive comparison, levodopa, selegiline, ropinirole and rotigotine for monotherapy in patients with Parkinson’s disease, and these results are in line with our results.

Patients with Parkinson’s disease are affected differently, and the need for pharmacological therapy varies for different ages and stages of the disease. As the treatment strategies are considered individually for each patient with Parkinson’s disease, it is reassuring for both clinicians and patients that the results from this MTC analysis indicate that all of the included dopamine agonists and MAO-B inhibitors, except safinamide, are effective compared with placebo. We found dopamine agonists, in particular ropinirole and pramipexole, to be effective and safe as monotherapy in managing symptoms of Parkinson’s disease. Although we found an increased risk of side effects related to pramipexole, we also found that there was no increased risk of withdrawal with this treatment, suggesting that the benefits from this treatment might outweigh the potential harms. Considering combination therapy, we found selegiline, ropinirole and pramipexole to be both effective and safe treatment options for these patients.

There are some limitations to this study. As with any MTC analysis, there is a potential weakness regarding the comparability of the included trials. Differences in patient demographics and the follow-up time might potentially introduce heterogeneity in the results. We adjusted for dose level, disease duration and duration of study, which will capture some of the possible differences, but other variables could also have been considered. However, considering too many variables could potentially lead to exclusion of too many trials due to lack of information, which again could introduce selection bias. Secondly, it is known that studies with positive findings are more likely to be published than studies with negative findings, giving a biased MTC analysis. We only had access to published studies, and that should be kept in mind when considering the results. With respect to our focus of ranking the drugs, we have no reason to believe that the publication bias was greater for some drugs than others.

In conclusion, we found dopamine agonists to be effective as treatment for Parkinson’s disease, both when given as monotherapy and in combination with levodopa, and the MAO-B inhibitor selegiline was found to be the best option when given in combination with levodopa. Treatment options must be individualized and tailored to the needs of each individual patient.

## Electronic supplementary material

ESM 1 (DOCX 802 kb)

## Data Availability

The data that supports the findings in this study are available in the supplementary material of this article (Appendix S8 and S9). The complete dataset is available from the researchers upon request.
