# Supplementary material for: Comparative effectiveness of dopamine agonists and monoamine oxidase type-B inhibitors for Parkinson’s disease: a multiple treatment comparison meta-analysis
Source: Eur J Clin Pharmacol. 2020 Jul 24;76(12):1731–43. doi: 10.1007/s00228-020-02961-6 (PMC7661406; doi:10.1007/s00228-020-02961-6)
Supplement: Supplementary file 1 — (DOCX 802 kb) [file 228_2020_2961_MOESM1_ESM.docx]

**Appendix S1**

**Search strategy in MEDLINE**

Database: Ovid MEDLINE(R) Epub Ahead of Print, In-Process & Other Non-Indexed

Citations, Ovid MEDLINE(R) Daily and Ovid MEDLINE(R) <1946 to Present>

Search Strategy:

--------------------------------------------------------------------------------

1 cabergoline.mp. (1556)

2 exp Parkinson Disease/ (58449)

3 1 and 2 (208)

4 limit 3 to (clinical trial, all or controlled clinical trial or randomized controlled trial)

(41)

Database: Ovid MEDLINE(R) Epub Ahead of Print, In-Process & Other Non-Indexed

Citations, Ovid MEDLINE(R) Daily and Ovid MEDLINE(R) <1946 to Present>

Search Strategy:

--------------------------------------------------------------------------------

1 apomorphine.mp. (12498)

2 exp Parkinson Disease/ (58482)

3 1 and 2 (1052)

4 limit 3 to (clinical trial, all or controlled clinical trial or randomized controlled trial)

(123)

Database: Ovid MEDLINE(R) Epub Ahead of Print, In-Process & Other Non-Indexed

Citations, Ovid MEDLINE(R) Daily and Ovid MEDLINE(R) <1946 to Present>

Search Strategy:

--------------------------------------------------------------------------------

1 pramipexole.mp. (1402)

2 exp Parkinson Disease/ (58425)

3 1 and 2 (541)

4 limit 3 to (clinical trial, all or controlled clinical trial or randomized controlled trial)

(116)

Database: Ovid MEDLINE(R) Epub Ahead of Print, In-Process & Other Non-Indexed

Citations, Ovid MEDLINE(R) Daily and Ovid MEDLINE(R) <1946 to Present>

Search Strategy:

--------------------------------------------------------------------------------

1 ropinirole.mp. (865)

2 exp Parkinson Disease/ (58425)

3 1 and 2 (392)

4 limit 3 to (clinical trial, all or controlled clinical trial or randomized controlled trial)

(86)

Database: Ovid MEDLINE(R) Epub Ahead of Print, In-Process & Other Non-Indexed

Citations, Ovid MEDLINE(R) Daily and Ovid MEDLINE(R) <1946 to Present>

Search Strategy:

--------------------------------------------------------------------------------

1 rotigotine.mp. (524)

2 exp Parkinson Disease/ (58490)

3 1 and 2 (195)

4 limit 3 to (clinical trial, all or controlled clinical trial or randomized controlled trial)

(57)

**Appendix S2**

**Addendum to the Protocol**

**Comparative effectiveness of pharmaceuticals for Parkinson’s disease**

The current work is part of a larger project examining Parkinson patients and their drug use from both clinical trials and Norwegian registers. Below is a detailed description on how we performed the current network meta-analysis based on published clinical trials.

*Objective*

To predict the safest and most optimal therapy for individual patients by analyzing and comprehensively comparing the effectiveness of Parkinson medication in different patient groups from all pooled international randomized controlled trials.

*Background*

A drug class review comparing MAO-B inhibitors and dopamine agonists will be prepared. This systematic review will be based on a comprehensive literature search and pooling of data from all published clinical trials involving the two drug classes. The network meta-analysis (NMA) will compare clinical effectiveness of both drug classes in a joint model. Both efficacy and safety endpoints will be assessed in the analysis. We will conduct an analysis in the lines of Tvete et al 2015 (1). All direct- and indirect clinical evidence will be pooled and analyzed separately for each individual endpoint. In this way we will be able to suggest which Parkinson’s drug treatment should be used as first-line and which should be used as second-line. We will also be able to give information regarding avoidable adverse events.

*Data sources*

The literature searches will be conducted using MEDLINE, PubMed and Cochrane Central Register of Controlled Trials to identify potentially eligible RCTs. We will include randomized controlled trials (RCTs) which examine UPDRS-total score, serious adverse events (SAEs), dropout or discontinuation of drug use in patients aged 18 or older with Parkinson’s disease. We are interested in the following interventions: *rasagiline, selegiline, safinamide, pramipexole, ropinirole, rotigotine or cabergoline* compared to placebo or any drug treatment. The drugs can be used either as monotherapy or in combination with each other. We will follow the methods outlined in the Cochrane handbook for systematic reviews of interventions (2). We will also look for ongoing trials in ClinicalTrials.gov and WHO International Clinical Trials Registry Platform (ICTRP).

*Eligibility criteria*

Population: Patients with Parkinson’s disease aged 18 or older

Interventions: MAO-B inhibitors (rasagiline, selegiline and safinamide) or dopamine agonists (pramipexole, ropinirole, rotigotine and cabergoline)

The above interventions can be used as monotherapy or in combination with each other or with levodopa.

Control: Placebo or any of the above mentioned interventions

Outcomes: Responders^#^ (primary endpoint)

Serious adverse events

Mortality

Withdrawals (discontinuation of drug use)

Need for levodopa

^#^ We define responders as the number of patients with at least 20 % reduction from baseline to end of study in the UPDRS score (total UPDRS score is used where this is provided, parts II and III or only part III where only these are provided), or an improvement (minimally improved, much improved or very much improved) on the Clinical Global Impression (CGI) scale.

*Data extraction and synthesis*

We will follow the methods outlined in the Cochrane Handbook for Systematic Reviews of Interventions (2). Two reviewers will independently inspect all citations to identify potentially relevant articles based on title and/or abstract. Full text publications will be obtained for articles appearing to meet our inclusion criteria. Two persons will independently assess whether the article is relevant or not according to our list of inclusion criteria. Disagreements will be resolved by discussion. One reviewer will extract the data from the included articles and another reviewer will check these results for accuracy. The studies will be assessed for risk of bias by using the Cochrane risk-of-bias tool for randomized trials on the following five domains: the randomization process, assignment to intervention, missing outcome data, measurement of the outcome and selection of the reported result. Studies assessed to have high risk of bias on more than two domains will not be included.

*Statistical analysis*

The statistical analyses will be conducted in the open source statistical software R (version 3.1.1, R Development Core Team). The statistical software R is a standard statistical software for conducting statistical analyses, commonly used by statisticians throughout the world. It is open source and free for anyone to use. We will construct a joint model for assessing the comparable relative effect between treatments (MAO-B inhibitor or dopamine agonists) across all trials, and perform a Bayesian statistical analysis based on this model inspired by Tvete et al (1). The model will take into consideration known background information (age, sex, duration of disease, duration of treatment, drug dose and if it is mono- or combination therapy). We will rank the treatments with respect to efficacy (change in total UPDRS score) and side effects (any serious adverse event and discontinuation of drug use). Taking such a modelling approach, we can report the probability that one treatment is better than another treatment and rank the different drugs.

References

1. Tvete IF, Natvig B, Gasemyr J, Meland N, Roine M, Klemp M. Comparing Effects of Biologic Agents in Treating Patients with Rheumatoid Arthritis: A Multiple Treatment Comparison Regression Analysis. PLoS One. 2015;10(9):e0137258.

2. Higgins JPT TJ, Chandler J, Cumpston M, Li T, Page MJ, Welch VA (editors). Cochrane Handbook for Systematic Reviews of Interventions version 6.0: Cochrane; 2019. Available from: [www.training.cochrane.org/handbook](file:///C:\Users\mklemp\AppData\Local\Microsoft\Windows\INetCache\Content.Outlook\JN0YNK31\www.training.cochrane.org\handbook).

**Appendix S3**

**Risk of bias assessment of included clinical trials**

**
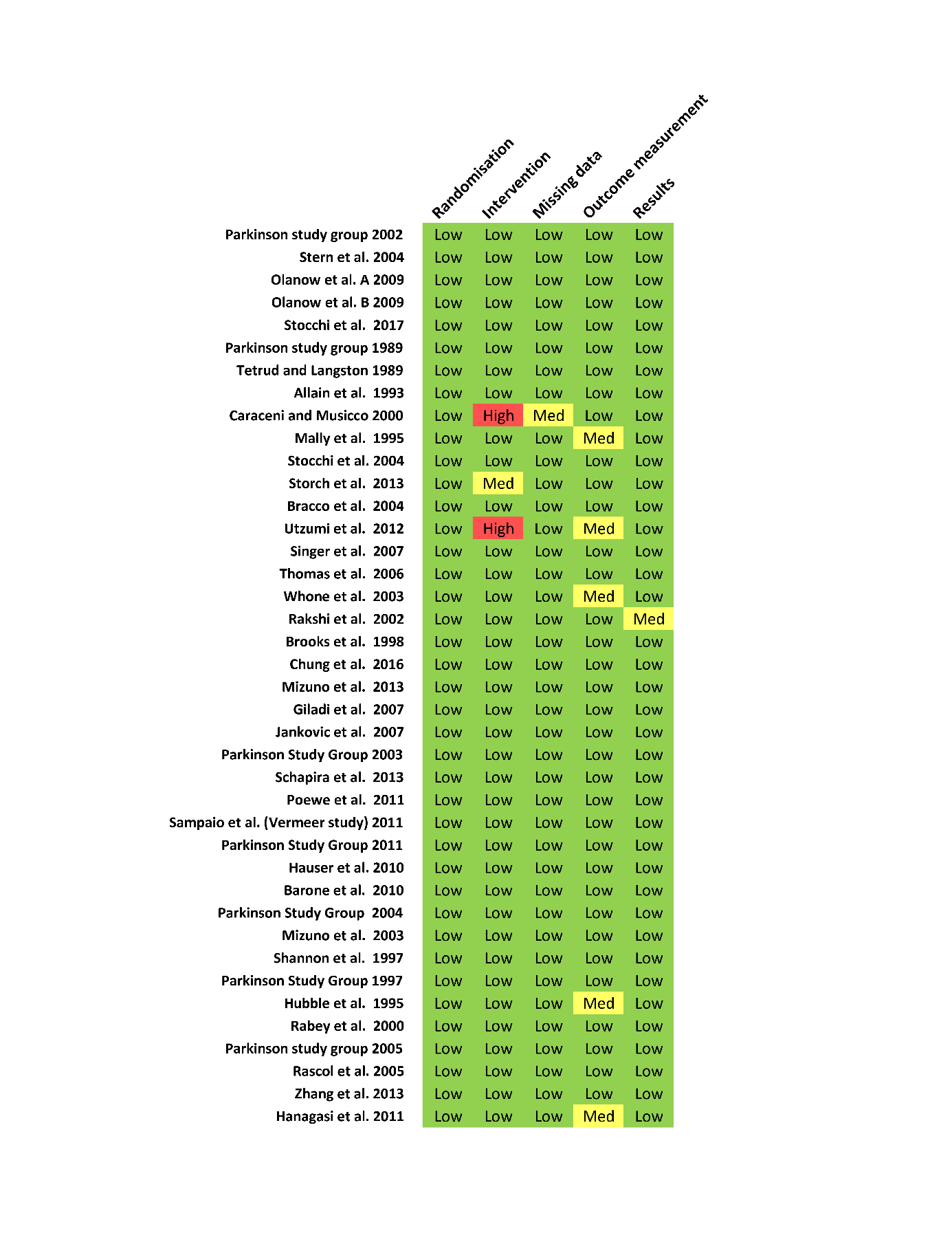
**

**
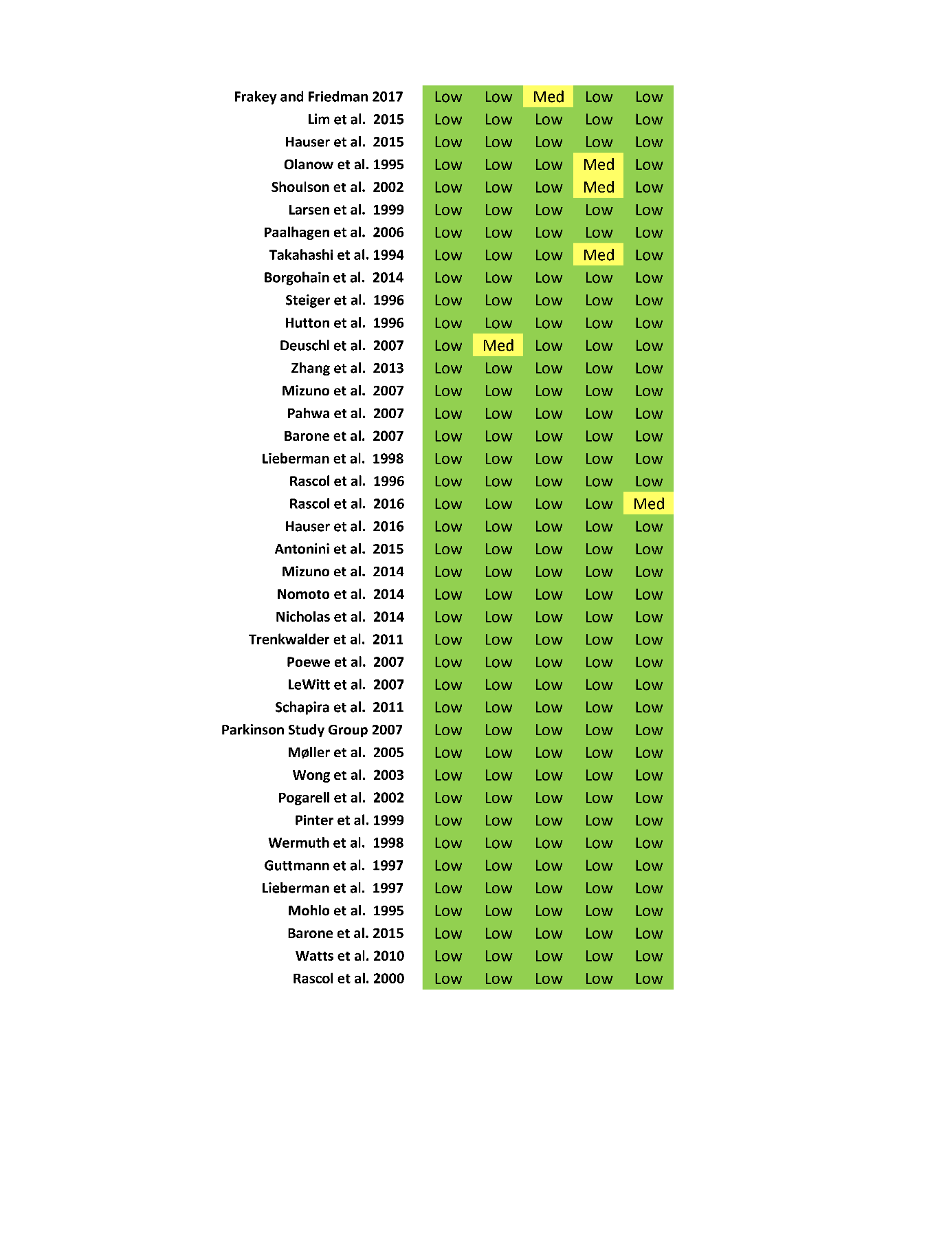
**

**Appendix S4**

**Individual cut-off levels for the different drugs**

|  | Dose | |
| --- | --- | --- |
|  | Low | High |
| Cabergoline | <3 mg/day | ≥3 mg/day |
| Ropinirole | <13 mg/day | ≥13 mg/day |
| Rotigotine | <8 mg/day | ≥8 mg/day |
| Pramipexole | <3.3 mg/day | ≥3.3 mg/day |
| Selegiline | <10 mg/day | ≥10 mg/day |
| Rasagiline | <1 mg/day | ≥1 mg/day |
| Safinamide | <100 mg/day | ≥100 mg/day |

**Appendix S5**

**The statistical model**

We will compare the effect, the degree of serious adverse events and withdrawals with respect to given drug treatment. We will first describe a model where we do not take into consideration the dose level, disease duration or study duration (basic model). We will then expand the model to also include these variables. We present the model for measuring the effect in network one. The model for network two, and for the two other endpoints serious adverse events and withdrawals, follows similarly.

**Basic model: comparing treatment with DA- or MAO-B drugs to placebo treatment**

We let r_ij_ denote the number of patients with effect in treatment arm j in study i, i =1, …, S, j =1, …, a_i_, where a_i_ is the number of arms in study i, and a_i_ varies between 2 and maximum 5 arms. We let m_ij_ denote the number of patients in study i in arm j. We let p_ki_ denote the probability in study i to achieve effect for treatment k, where k is one of the nine treatments: RA, SA, SE, CAB, PRA, ROP, ROT, LD or placebo (P).

We let r_ij_ follow a binomial distribution. We assume that the multiplicative treatment effects relative to placebo are given by effect-ratios γ^k,p^=p_ki_/p_pi_, where k denotes treatment with RA, SA, SE, CAB, PRA, ROP, ROT or LD (k is hence one of eight treatments, not placebo). We assume that effect-ratios are constant over studies.

Let $A_{i}$ denote the set of treatment arms in study i. Let k_j_ denote the treatment given in arm j. The likelihood is:

$\prod_{i=1}^{S}\prod_{{jA}_{i}} {(p_{pi} {}^{k_{j},p})}^{r_{ij}}$(1 - ${p_{pi} {}^{k_{j},p})}^{{m_{ij}- r}_{ij}}$_._

We assume that γ^k,p^_~_ lognormal(0,σ^2^_φ_), k= RA, SA, SE, CAB, PRA, ROP, ROT or LD and σ_φ_~uniform(0,20).

Parameters estimated in the model are the p_pi_s, the γs and σ_φ_. We assume that p_pi_, i =1, …, S, is uniformly distributed, with an upper limit dependent upon the γs which enters study i in the following way:

$p_{pi}\sim uniform(0,min(1,{min}_{{jA}_{i}}1/{}^{k_{j},p}))$.

We focus on the effect of RA, SA, SE, CAB, PRA, ROP, ROT or LD treatment compared to placebo.

**Advanced model: considering dose level, disease duration and study duration**

We incorporate dose level, disease duration and study duration into the model by letting the multiplicative treatment effect depend upon dose level (X^DL^), disease duration (X^DD^) and study duration (X^SD^):

$G_{ij}= {}^{k_{j},p} {exp}^{\beta_{DL}X^{DL}+\beta_{DD}X^{DD}+\beta_{SD}X^{SD}}$.

The likelihood becomes:

$\prod_{i=1}^{S}\prod_{{jA}_{i}} {(p_{pi} G_{ij})}^{r_{ij}}$(1 - ${p_{pi} G_{ij})}^{{m_{ij}- r}_{ij}}$_,_ where

$p_{pi}\sim uniform(0,min(1,{min}_{{jA}_{i}}1/G_{ij}))$.

The placebo-probabilities p_pi_ are a priori uniformly distributed over a range dependent upon dose level, disease- and study duration. We let β_DL_ ~ normal(0,σ_DL_^2^), β_DD_ ~ normal(0,σ_DD_^2^) and β_SD_ ~ normal(0,σ_SD_^2^). We assume a priori that σ_DL_, σ_DD_ and σ_SD_ are uniformly distributed over (0,20).

In this Bayesian model, we construct probability distributions for the parameters of interest (the p_pi_s, the γs). The parameters are not to be interpreted as random variables, but our knowledge about them is uncertain, and we describe this uncertainty through probability distributions. We express our initial uncertainty in the prior distributions (prior to seeing data). Seeing the data the prior distributions update to posterior distributions by using Bayes formula. We give all parameter estimates with a corresponding credibility (uncertainty) interval.

The model was fitted in OpenBUGS run from R. Taking 1 000 000 burn-in iterations (that were disregarded) and thereafter another 1 000 000 new iterations where every 100 iteration was kept gave 10 000 samples from the full conditional distribution for each of the parameters. This formed the posterior distribution for the parameters of interest. We examined the MCMC chains for the parameters to check for convergence issues.

**Appendix S6**

**Excluded studies and reason for exclusion**

|  | Study |  | Reason for exclusion^a^ |
| --- | --- | --- | --- |
| 1 | Brusa et al. | 2013 | Wrong endpoint |
| 2 | Ahlskog et al. | 1996 | Wrong endpoint |
| 3 | Hutton et al. | 1993 | Wrong comparator |
| 4 | Odin et al. | 2006 | No control group |
| 5 | Högl et al. | 2003 | No control group |
| 6 | Rinne et al. | 1998 | Data from already included trial (Bracco 2004) |
| 7 | Del Dotto et al. | 1997 | Wrong comparator |
| 8 | Rinne et al. | 1997 | Data from already included trial (Bracco 2004) |
| 9 | Lieberman et al. | 1993 | No control group |
| 10 | Inzelberg et al. | 1996 | Wrong comparator |
| 11 | Chaudhuri et al. | 2012 | Post-hoc analysis of already included trial (Pahwa 2007) |
| 12 | Rektorova et al. | 2008 | Wrong comparator |
| 13 | Tompson et al. | 2007 | Wrong comparator, wrong endpoint |
| 14 | Hauser et al. | 2007 | Extension of already included trial (Rascol 2000) |
| 15 | Morgan and Sethi | 2004 | Data from already included trial (Whone 2003) |
| 16 | Rascol et al. | 2006 | Post-hoc analysis of already included trial (Rascol 2000) |
| 17 | Badarny et al. | 2006 | Wrong comparator |
| 18 | Müngersdorf et al. | 2001 | Wrong comparator |
| 19 | Korczyn et al. | 1998 | Data from already included trial (Korczyn 1999) |
| 20 | Rascol et al. | 1998 | Data from already included trial (Rascol 2000) |
| 21 | Stocchi et.al | 2008 | Wrong comparator |
| 22 | Stocchi et.al | 2011 | Wrong comparator |
| 23 | Tompson et al. | 2009 | Wrong comparator |
| 24 | Im et al. | 2003 | Wrong comparator |
| 25 | Brunt et al. | 2002 | Wrong comparator |
| 26 | Korczyn et al. | 1999 | Wrong comparator |
| 27 | Adler et al. | 1997 | Wrong intervention |
| 28 | Sethi et al. | 1998 | Extension of already included trial (Adler 1997) |
| 29 | Rascol et al. | 1996 | Data from already included trial (Rascol 1996) |
| 30 | Timmermann et al. | 2017 | Wrong comparator |
| 31 | Giladi et al. | 2014 | Extension of already included trials (Jancovic 2007 and Giladi 2007) |
| 32 | Garcia-Ruiz et al. | 2014 | Wrong comparator |
| 33 | Morgan and Sethi | 2004 | Data from already included trial (Parkinson study group 2003) |
| 34 | Watts et al. | 2007 | Data from already included trial (Jankovic 2007) |
| 35 | Hutton et al. | 2001 | Wrong endpoint |
| 36 | Eggert et al. | 2014 | Wrong endpoint |
| 37 | Hauser et al. | 2014 | Extension of already included trials (Schapira 2011 and Poewe 2011) |
| 38 | Utsumi et al. | 2013 | Wrong comparator |
| 39 | Schapira et al. | 2013 | Open label extension of already included trial (Schapira 2011) |
| 40 | Schapira et al. | 2013 | Wrong endpoint |
| 41 | Drijgers et al. | 2012 | Wrong comparator |
| 42 | Rascol et al. | 2010 | Wrong comparator |
| 43 | Schapira et al. | 2010 | Data on already included trial (Schapira 2013) |
| 44 | Brodsky et al. | 2010 | Wrong endpoint |
| 45 | Parkinson Study Group | 2009 | Follow-up on already included trial (Parkinson Study Group 2004) |
| 46 | Costa et al. | 2009 | Wrong comparator |
| 47 | Micallef et al. | 2009 | Wrong comparator |
| 48 | Biglan et al. | 2007 | Data from already included trial (Parkinson Study Group 2004) |
| 49 | Hauser et al. | 2006 | Data from already included trial (Parkinson Study Group 2004) |
| 50 | Barone et al. | 2006 | Wrong comparator |
| 51 | Noyes et al. | 2006 | Data from already included trial (Parkinson Study Group 2004), (Quality of Life endpoints) |
| 52 | Noyes et al. | 2005 | Data from already included trial (Parkinson Study Group 2004), (Cost Effectiveness) |
| 53 | Rektorova et al. | 2005 | Data from already included trial (Rektorova 2003) |
| 54 | Noyes et al. | 2004 | Data from already included trial (Parkinson Study Group 2004), (Cost effectiveness) |
| 55 | Rektorova et al. | 2003 | Wrong endpoint |
| 56 | Brusa et al. | 2003 | Wrong endpoint |
| 57 | Parkinson study group | 2002 | Substudy of already included trial (Parkinson Study Group 2004) |
| 58 | Parkinson study group | 2000 | Data from already included trial (Parkinson Study Group 2004) |
| 59 | Parkinson study group | 2000 | Data from already included trial (Parkinson Study Group 2004) |
| 60 | Künig et al. | 1999 | Wrong endpoint |
| 61 | Navan et al. | 2005 | Wrong comparator |
| 62 | Navan et al. | 2003 | Wrong endpoint |
| 63 | Olanow et al. | 2017 | Wrong comparator |
| 64 | Mizuno et al. | 2012 | Wrong comparator |
| 65 | Navan et al. | 2003 | Wrong endpoint |

^a^Reasons for exclusion are given according to PICO (P, population; I, Intervention; C, comparator; O, outcome/endpoint)

**Appendix S7**

**Overview of included studies and treatment arms**

| **Network** |  | **Publication** | **Control** | **Treat_arm1_** | **Treat_arm2_** | **Treat_arm3_** | **Treat_arm4_** |
| --- | --- | --- | --- | --- | --- | --- | --- |
| 1 | 1 | Parkinson study group 2002 | P | RA | RA |  |  |
|  | 2 | Stern et al. 2004 | P | RA | RA | RA |  |
|  | 3A | Olanow et al. A 2009 | P | RA |  |  |  |
|  | 3B | Olanow et al. B 2009 | P | RA |  |  |  |
|  | 4 | Stocchi et al. 2017 | P | RA |  |  |  |
|  | 5 | Parkinson study group 1989 | P | SE |  |  |  |
|  | 6 | Tetrud and Langston 1989 | P | SE |  |  |  |
|  | 7 | Allain et al. 1993 | P | SE |  |  |  |
|  | 8 | Caraceni and Musicco 2000 | LD | SE |  |  |  |
|  | 9 | Mally et al. 1995 | P | SE |  |  |  |
|  | 10 | Stocchi et al. 2004 | P | SA | SA |  |  |
|  | 11 | Storch et al. 2013 | LD | CAB |  |  |  |
|  | 12 | Bracco et al. 2004 | LD | CAB |  |  |  |
|  | 13 | Utzumi et al. 2012 | LD | CAB |  |  |  |
|  | 14 | Singer et al. 2007 | P | ROP |  |  |  |
|  | 15 | Thomas et al. 2006 | PRA | ROP |  |  |  |
|  | 16 | Whone et al. 2003 | LD | ROP |  |  |  |
|  | 17 | Rakshi et al. 2002 | LD | ROP |  |  |  |
|  | 18 | Brooks et al. 1998 | P | ROP |  |  |  |
|  | 19 | Chung et al. 2016 | P | ROT |  |  |  |
|  | 20 | Mizuno et al. 2013 | P | ROT |  |  |  |
|  | 21 | Giladi et al. 2007 | P | ROT | ROP |  |  |
|  | 22 | Jankovic et al. 2007 | P | ROT |  |  |  |
|  | 23 | Parkinson Study Group 2003 | P | ROT | ROT | ROT | ROT |
|  | 24 | Schapira et al. 2013 | P | PRA |  |  |  |
|  | 25 | Poewe et al. 2011 | P | PRA | PRA |  |  |
|  | 26 | Sampaio et al. (Vermeer study) 2011 | P | PRA |  |  |  |
|  | 27 | Parkinson Study Group 2011 | P | PRA | PRA | PRA |  |
|  | 28 | Hauser et al. 2010 | P | PRA | PRA |  |  |
|  | 29 | Barone et al. 2010 | P | PRA |  |  |  |
|  | 30 | Parkinson Study Group 2004 | LD | PRA |  |  |  |
|  | 31 | Mizuno et al. 2003 | P | PRA |  |  |  |
|  | 32 | Shannon et al. 1997 | P | PRA |  |  |  |
|  | 33 | Parkinson Study Group 1997 | P | PRA | PRA | PRA | PRA |
|  | 34 | Hubble et al. 1995 | P | PRA |  |  |  |
| 2 | 35 | Rabey et al. 2000 | P+LD | RA+LD | RA+LD | RA+LD |  |
|  | 36 | Parkinson study group 2005 | P+LD | RA+LD | RA+LD |  |  |
|  | 37 | Rascol et al. 2005 | P+LD | RA+LD | EN+LD |  |  |
|  | 38 | Zhang et al. 2013 | P+LD | RA+LD |  |  |  |
|  | 39 | Hanagasi et al. 2011 | P+LD | RA+LD |  |  |  |
|  | 40 | Frakey and Friedman 2017 | P+LD | RA+LD |  |  |  |
|  | 41 | Lim et al. 2015 | P+LD | RA+LD |  |  |  |
|  | 42 | Hauser et al. 2015 | P+LD | RA+LD |  |  |  |
|  | 43 | Olanow et al. 1995 | P+LD | SE+LD |  |  |  |
|  | 44 | Shoulson et al. 2002 | P+LD | SE+LD |  |  |  |
|  | 45 | Larsen et al. 1999 | P+LD | SE+LD |  |  |  |
|  | 46 | Paalhagen et al. 2006 | P+LD | SE+LD |  |  |  |
|  | 47 | Takahashi et al. 1994 | P+LD | SE+LD |  |  |  |
|  | 48 | Borgohain et al. 2014 | P+LD | SA+LD | SA+LD |  |  |
|  | 49 | Steiger et al. 1996 | P+LD | CAB+LD |  |  |  |
|  | 50 | Hutton et al. 1996 | P+LD | CAB+LD |  |  |  |
|  | 51 | Deuschl et al. 2007 | EN+LD | CAB+LD |  |  |  |
|  | 52 | Zhang et al. 2013 | P+LD | ROP+LD |  |  |  |
|  | 53 | Mizuno et al. 2007 | P+LD | ROP+LD |  |  |  |
|  | 54 | Pahwa et al. 2007 | P+LD | ROP+LD |  |  |  |
|  | 55 | Barone et al. 2007 | P+LD | ROP+LD |  |  |  |
|  | 56 | Lieberman et al. 1998 | P+LD | ROP+LD |  |  |  |
|  | 57 | Rascol et al. 1996 | P+LD | ROP+LD |  |  |  |
|  | 58 | Watts et al. 2010 | LD | ROP+LD |  |  |  |
|  | 59 | Rascol et al. 2000 | LD | ROP+LD |  |  |  |
|  | 60 | Rascol et al. 2016 | P+LD | ROT+LD |  |  |  |
|  | 61 | Hauser et al. 2016 | P+LD | ROT+LD | ROT+LD |  |  |
|  | 62 | Antonini et al. 2015 | P+LD | ROT+LD |  |  |  |
|  | 63 | Mizuno et al. 2014 | P+LD | ROT+LD | ROP+LD |  |  |
|  | 64 | Nomoto et al. 2014 | P+LD | ROT+LD |  |  |  |
|  | 65 | Nicholas et al. 2014 | P+LD | ROT+LD | ROT+LD | ROT +LD | ROT+LD |
|  | 66 | Trenkwalder et al. 2011 | P+LD | ROT+LD |  |  |  |
|  | 67 | Poewe et al. 2007 | P+LD | ROT+ LD | PRA+LD |  |  |
|  | 68 | LeWitt et al. 2007 | P+LD | ROT+LD | ROT+LD |  |  |
|  | 69 | Schapira et al. 2011 | P+LD | PRA+LD | PRA+LD |  |  |
|  | 70 | Parkinson Study Group 2007 | P+LD | PRA+LD |  |  |  |
|  | 71 | Møller et al. 2005 | P+LD | PRA+LD |  |  |  |
|  | 72 | Wong et al. 2003 | P+LD | PRA+LD |  |  |  |
|  | 73 | Pogarell et al. 2002 | P+LD | PRA+LD |  |  |  |
|  | 74 | Pinter et al. 1999 | P+LD | PRA+LD |  |  |  |
|  | 75 | Wermuth et al. 1998 | P+LD | PRA+LD |  |  |  |
|  | 76 | Guttmann et al. 1997 | P+LD | PRA+LD |  |  |  |
|  | 77 | Lieberman et al. 1997 | P+LD | PRA+LD |  |  |  |
|  | 78 | Mohlo et al. 1995 | P+LD | PRA +LD |  |  |  |
|  | 79 | Barone et al. 2015 | P+LD | RA+LD |  |  |  |

RA, rasagiline; SA, safinamide; SE, selegiline; CAB, cabergoline; PRA, pramipexole; ROP, ropinirole; ROT, rotigotine; LD, levodopa; EN, entacapone

**Appendix S8**

**Number of patients, responders, serious adverse events and withdrawals in network 1**

|  |  | Number | | | | | Effect | | | | | SAE | | | | | Withdrawals | | | | |
| --- | --- | --- | --- | --- | --- | --- | --- | --- | --- | --- | --- | --- | --- | --- | --- | --- | --- | --- | --- | --- | --- |
| Number | Study | C | T1 | T2 | T3 | T4 | C | T1 | T2 | T3 | T4 | C | T1 | T2 | T3 | T4 | C | T1 | T2 | T3 | T4 |
| 1 | Parkinson study group 2002 | 138 | 134 | 132 | 0 | 0 | 68 | 85 | 88 | 0 | 0 | 4 | 6 | 10 | 0 | 0 | 3 | 8 | 5 | 0 | 0 |
| 2 | Stern et al. 2004 | 13 | 15 | 14 | 14 | 0 | 0 | 5 | 4 | 4 | 0 | 0 | 0 | 0 | 0 | 0 | 0 | 1 | 0 | 0 | 0 |
| 3 | Olanow et al. A 2009 | 300 | 288 | 0 | 0 | 0 | 111 | 202 | 0 | 0 | 0 | 0 | 1 | 0 | 0 | 0 | 30 | 15 | 0 | 0 | 0 |
| 4 | Olanow et al. B 2009 | 295 | 293 | 0 | 0 | 0 | 109 | 217 | 0 | 0 | 0 | 0 | 0 | 0 | 0 | 0 | 20 | 20 | 0 | 0 | 0 |
| 5 | Stocchi et al. 2017 | 204 | 204 | 0 | 0 | 0 | 72 | 68 | 0 | 0 | 0 | 7 | 6 | 0 | 0 | 0 | 19 | 15 | 0 | 0 | 0 |
| 6 | Parkinson study group 1989 | 401 | 399 | 0 | 0 | 0 | 132 | 215 | 0 | 0 | 0 | 6 | 5 | 0 | 0 | 0 | 24 | 24 | 0 | 0 | 0 |
| 7 | Tetrud and Langston 1989 | 27 | 27 | 0 | 0 | 0 | 9 | 18 | 0 | 0 | 0 | 2 | 0 | 0 | 0 | 0 | 5 | 5 | 0 | 0 | 0 |
| 8 | Allain et al. 1993 | 45 | 48 | 0 | 0 | 0 | 31 | 43 | 0 | 0 | 0 | 0 | 0 | 0 | 0 | 0 | 6 | 3 | 0 | 0 | 0 |
| 9 | Caraceni and Musicco 2000 | 156 | 155 | 0 | 0 | 0 | 113 | 104 | 0 | 0 | 0 | 0 | 0 | 0 | 0 | 0 | 10 | 30 | 0 | 0 | 0 |
| 10 | Mally et al. 1995 | 10 | 10 | 0 | 0 | 0 | 1 | 5 | 0 | 0 | 0 | 0 | 0 | 0 | 0 | 0 | 0 | 0 | 0 | 0 | 0 |
| 11 | Stocchi et al. 2004 | 56 | 56 | 56 | 0 | 0 | 12 | 17 | 21 | 0 | 0 | 0 | 1 | 0 | 0 | 0 | 7 | 4 | 7 | 0 | 0 |
| 12 | Storch et al. 2013 | 20 | 19 | 0 | 0 | 0 | 7 | 6 | 0 | 0 | 0 | 0 | 0 | 0 | 0 | 0 | 2 | 2 | 0 | 0 | 0 |
| 13 | Bracco et al. 2004 | 209 | 211 | 0 | 0 | 0 | 142 | 100 | 0 | 0 | 0 | 47 | 58 | 0 | 0 | 0 | 79 | 88 | 0 | 0 | 0 |
| 14 | Utzumi et al. OPEN LABEL 2012 | 49 | 49 | 0 | 0 | 0 | 15 | 10 | 0 | 0 | 0 | 2 | 6 | 0 | 0 | 0 | 22 | 37 | 0 | 0 | 0 |
| 15 | Singer et al. 2007 | 205 | 205 | 0 | 0 | 0 | 51 | 105 | 0 | 0 | 0 | 12 | 17 | 0 | 0 | 0 | 108 | 95 | 0 | 0 | 0 |
| 16 | Thomas et al. 2006 | 30 | 30 | 0 | 0 | 0 | 8 | 8 | 0 | 0 | 0 | 0 | 0 | 0 | 0 | 0 | 5 | 3 | 0 | 0 | 0 |
| 17 | Whone et al. 2003 | 93 | 93 | 0 | 0 | 0 | 56 | 59 | 0 | 0 | 0 | 17 | 18 | 0 | 0 | 0 | 20 | 23 | 0 | 0 | 0 |
| 18 | Rakshi et al. 2002 | 14 | 31 | 0 | 0 | 0 | 6 | 14 | 0 | 0 | 0 | 0 | 0 | 0 | 0 | 0 | 5 | 3 | 0 | 0 | 0 |
| 19 | Brooks et al. 1998 | 22 | 41 | 0 | 0 | 0 | 9 | 29 | 0 | 0 | 0 | 2 | 3 | 0 | 0 | 0 | 3 | 5 | 0 | 0 | 0 |
| 20 | Chung et al. 2016 | 196 | 184 | 0 | 0 | 0 | 20 | 31 | 0 | 0 | 0 | 21 | 9 | 0 | 0 | 0 | 32 | 35 | 0 | 0 | 0 |
| 21 | Mizuno et al. 2013 | 90 | 90 | 0 | 0 | 0 | 36 | 63 | 0 | 0 | 0 | 0 | 2 | 0 | 0 | 0 | 10 | 15 | 0 | 0 | 0 |
| 22 | Giladi et al. 2007 | 118 | 215 | 228 | 0 | 0 | 35 | 112 | 155 | 0 | 0 | 10 | 21 | 30 | 0 | 0 | 41 | 75 | 60 | 0 | 0 |
| 23 | Jankovic et al. 2007 | 96 | 181 | 0 | 0 | 0 | 18 | 87 | 0 | 0 | 0 | 2 | 14 | 0 | 0 | 0 | 15 | 39 | 0 | 0 | 0 |
| 24 | Parkinson Study Group 2003 | 47 | 49 | 47 | 48 | 51 | 5 | 8 | 12 | 17 | 21 | 1 | 3 | 0 | 3 | 2 | 7 | 8 | 4 | 9 | 8 |
| 25 | Schapira et al. 2013 | 274 | 261 | 0 | 0 | 0 | 69 | 130 | 0 | 0 | 0 | 18 | 17 | 0 | 0 | 0 | 60 | 40 | 0 | 0 | 0 |
| 26 | Poewe et al. 2011 | 103 | 223 | 213 | 0 | 0 | 36 | 142 | 132 | 0 | 0 | 4 | 16 | 11 | 0 | 0 | 12 | 49 | 37 | 0 | 0 |
| 27 | Sampaio et al. (Vermeer study) 2011 | 110 | 116 | 0 | 0 | 0 | 48 | 73 | 0 | 0 | 0 | 0 | 12 | 0 | 0 | 0 | 22 | 30 | 0 | 0 | 0 |
| 28 | Parkinson Study Group 2011 | 77 | 81 | 73 | 80 | 0 | 21 | 40 | 36 | 39 | 0 | 0 | 0 | 2 | 1 | 0 | 5 | 11 | 11 | 10 | 0 |
| 29 | Hauser et al. 2010 | 50 | 106 | 103 | 0 | 0 | 8 | 39 | 49 | 0 | 0 | 1 | 5 | 3 | 0 | 0 | 4 | 21 | 15 | 0 | 0 |
| 30 | Barone et al. 2010 | 152 | 144 | 0 | 0 | 0 | 32 | 46 | 0 | 0 | 0 | 6 | 6 | 0 | 0 | 0 | 19 | 20 | 0 | 0 | 0 |
| 31 | Parkinson Study Group 2004 | 150 | 151 | 0 | 0 | 0 | 61 | 32 | 0 | 0 | 0 | 2 | 5 | 0 | 0 | 0 | 50 | 68 | 0 | 0 | 0 |
| 32 | Mizuno et al. 2003 | 108 | 102 | 0 | 0 | 0 | 39 | 63 | 0 | 0 | 0 | 0 | 3 | 0 | 0 | 0 | 15 | 13 | 0 | 0 | 0 |
| 33 | Shannon et al. 1997 | 171 | 164 | 0 | 0 | 0 | 26 | 82 | 0 | 0 | 0 | 8 | 18 | 0 | 0 | 0 | 34 | 28 | 0 | 0 | 0 |
| 34 | Parkinson Study Group 1997 | 51 | 54 | 50 | 54 | 55 | 14 | 27 | 25 | 26 | 28 | 0 | 1 | 1 | 3 | 5 | 0 | 10 | 2 | 4 | 9 |
| 35 | Hubble et al. 1995 | 27 | 28 | 0 | 0 | 0 | 11 | 19 | 0 | 0 | 0 | 1 | 8 | 0 | 0 | 0 | 1 | 0 | 0 | 0 | 0 |

(C: control arm, T1-4: treatment arm 1-4)

**Appendix S9**

**Number of patients, responders, serious adverse events and withdrawals in network 2**

|  |  | Number | | | | | Effect | | | | | SAE | | | | | Withdrawals | | | | |
| --- | --- | --- | --- | --- | --- | --- | --- | --- | --- | --- | --- | --- | --- | --- | --- | --- | --- | --- | --- | --- | --- |
| Number | Study | C | T1 | T2 | T3 | T4 | C | T1 | T2 | T3 | T4 | C | T1 | T2 | T3 | T4 | C | T1 | T2 | T3 | T4 |
| 1 | Rabey et al. 2000 | 13 | 21 | 18 | 18 | 0 | 5 | 12 | 10 | 10 | 0 | 1 | 0 | 0 | 0 | 0 | 1 | 1 | 3 | 1 | 0 |
| 2 | Parkinson study group 2005 | 159 | 164 | 149 | 0 | 0 | 24 | 38 | 44 | 0 | 0 | 14 | 21 | 18 | 0 | 0 | 19 | 22 | 17 | 0 | 0 |
| 3 | Rascol et al. 2005 | 229 | 231 | 227 | 0 | 0 | 70 | 113 | 99 | 0 | 0 | 17 | 12 | 12 | 0 | 0 | 35 | 23 | 30 | 0 | 0 |
| 4 | Zhang et al. 2013 | 125 | 119 | 0 | 0 | 0 | 37 | 63 | 0 | 0 | 0 | 1 | 1 | 0 | 0 | 0 | 14 | 11 | 0 | 0 | 0 |
| 5 | Hanagasi et al. 2011 | 25 | 23 | 0 | 0 | 0 | 5 | 10 | 0 | 0 | 0 | 0 | 0 | 0 | 0 | 0 | 0 | 0 | 0 | 0 | 0 |
| 6 | Frakey and Friedman 2017 | 22 | 23 | 0 | 0 | 0 | 4 | 11 | 0 | 0 | 0 | 0 | 0 | 0 | 0 | 0 | 0 | 0 | 0 | 0 | 0 |
| 7 | Lim et al. 2015 | 14 | 16 | 0 | 0 | 0 | 2 | 8 | 0 | 0 | 0 | 0 | 0 | 0 | 0 | 0 | 0 | 0 | 0 | 0 | 0 |
| 8 | Hauser et al. 2015 | 155 | 156 | 0 | 0 | 0 | 52 | 56 | 0 | 0 | 0 | 6 | 9 | 0 | 0 | 0 | 21 | 27 | 0 | 0 | 0 |
| 9 | Olanow et al. 1995 | 49 | 52 | 0 | 0 | 0 | 5 | 18 | 0 | 0 | 0 | 0 | 0 | 0 | 0 | 0 | 9 | 10 | 0 | 0 | 0 |
| 10 | Shoulson et al. 2002 | 177 | 191 | 0 | 0 | 0 | 18 | 52 | 0 | 0 | 0 | 13 | 15 | 0 | 0 | 0 | 48 | 42 | 0 | 0 | 0 |
| 11 | Larsen et al. 1999 | 81 | 73 | 0 | 0 | 0 | 0 | 22 | 0 | 0 | 0 | 3 | 8 | 0 | 0 | 0 | 24 | 28 | 0 | 0 | 0 |
| 12 | Paalhagen et al. 2006 | 69 | 72 | 0 | 0 | 0 | 12 | 36 | 0 | 0 | 0 | 2 | 4 | 0 | 0 | 0 | 30 | 29 | 0 | 0 | 0 |
| 13 | Takahashi et al. 1994 | 52 | 60 | 0 | 0 | 0 | 24 | 44 | 0 | 0 | 0 | 0 | 1 | 0 | 0 | 0 | 5 | 7 | 0 | 0 | 0 |
| 14 | Borgohain et al. 2014 | 222 | 223 | 224 | 0 | 0 | 123 | 148 | 144 | 0 | 0 | 28 | 32 | 34 | 0 | 0 | 27 | 38 | 25 | 0 | 0 |
| 15 | Steiger et al. 1996 | 18 | 19 | 0 | 0 | 0 | 6 | 11 | 0 | 0 | 0 | 0 | 1 | 0 | 0 | 0 | 0 | 0 | 0 | 0 | 0 |
| 16 | Hutton et al. 1996 | 65 | 123 | 0 | 0 | 0 | 4 | 20 | 0 | 0 | 0 | 5 | 8 | 0 | 0 | 0 | 11 | 13 | 0 | 0 | 0 |
| 17 | Deuschl et al. OPEN LABEL 2007 | 82 | 79 | 0 | 0 | 0 | 21 | 20 | 0 | 0 | 0 | 6 | 3 | 0 | 0 | 0 | 13 | 13 | 0 | 0 | 0 |
| 18 | Zhang et al. 2013 | 171 | 176 | 0 | 0 | 0 | 16 | 77 | 0 | 0 | 0 | 8 | 9 | 0 | 0 | 0 | 35 | 12 | 0 | 0 | 0 |
| 19 | Mizuno et al. 2007 | 122 | 121 | 0 | 0 | 0 | 34 | 67 | 0 | 0 | 0 | 3 | 6 | 0 | 0 | 0 | 26 | 23 | 0 | 0 | 0 |
| 20 | Pahwa et al. 2007 | 191 | 202 | 0 | 0 | 0 | 27 | 83 | 0 | 0 | 0 | 7 | 8 | 0 | 0 | 0 | 57 | 34 | 0 | 0 | 0 |
| 21 | Barone et al. 2007 | 316 | 316 | 0 | 0 | 0 | 32 | 95 | 0 | 0 | 0 | 4 | 9 | 0 | 0 | 0 | 123 | 80 | 0 | 0 | 0 |
| 22 | Lieberman et al. 1998 | 54 | 95 | 0 | 0 | 0 | 17 | 56 | 0 | 0 | 0 | 0 | 0 | 0 | 0 | 0 | 22 | 21 | 0 | 0 | 0 |
| 23 | Rascol et al. 1996 | 23 | 23 | 0 | 0 | 0 | 8 | 18 | 0 | 0 | 0 | 3 | 5 | 0 | 0 | 0 | 9 | 2 | 0 | 0 | 0 |
| 24 | Rascol et al. 2016 | 33 | 35 | 0 | 0 | 0 | 14 | 18 | 0 | 0 | 0 | 1 | 2 | 0 | 0 | 0 | 6 | 6 | 0 | 0 | 0 |
| 25 | Hauser et al. 2016 | 40 | 41 | 41 | 0 | 0 | 4 | 11 | 10 | 0 | 0 | 4 | 2 | 1 | 0 | 0 | 8 | 11 | 4 | 0 | 0 |
| 26 | Antonini et al. 2015 | 125 | 224 | 0 | 0 | 0 | 21 | 45 | 0 | 0 | 0 | 4 | 8 | 0 | 0 | 0 | 22 | 44 | 0 | 0 | 0 |
| 27 | Mizuno et al. 2014 | 85 | 168 | 167 | 0 | 0 | 31 | 112 | 96 | 0 | 0 | 6 | 7 | 5 | 0 | 0 | 17 | 26 | 23 | 0 | 0 |
| 28 | Nomoto et al. 2014 | 87 | 87 | 0 | 0 | 0 | 25 | 55 | 0 | 0 | 0 | 4 | 6 | 0 | 0 | 0 | 14 | 12 | 0 | 0 | 0 |
| 29 | Nicholas et al. 2014 | 108 | 101 | 107 | 104 | 94 | 18 | 21 | 27 | 21 | 32 | 5 | 7 | 5 | 8 | 0 | 27 | 22 | 23 | 22 | 14 |
| 30 | Trenkwalder et al. 2011 | 97 | 190 | 0 | 0 | 0 | 13 | 48 | 0 | 0 | 0 | 5 | 10 | 0 | 0 | 0 | 17 | 24 | 0 | 0 | 0 |
| 31 | Poewe et al. 2007 | 101 | 204 | 201 | 0 | 0 | 35 | 122 | 135 | 0 | 0 | 9 | 19 | 15 | 0 | 0 | 26 | 23 | 30 | 0 | 0 |
| 32 | LeWitt et al. 2007 | 120 | 120 | 111 | 0 | 0 | 41 | 68 | 61 | 0 | 0 | 0 | 0 | 0 | 0 | 0 | 28 | 33 | 30 | 0 | 0 |
| 33 | Schapira et al. 2011 | 178 | 165 | 175 | 0 | 0 | 70 | 103 | 118 | 0 | 0 | 6 | 5 | 7 | 0 | 0 | 21 | 19 | 12 | 0 | 0 |
| 34 | Parkinson Study Group 2007 | 35 | 109 | 0 | 0 | 0 | 7 | 35 | 0 | 0 | 0 | 0 | 3 | 0 | 0 | 0 | 1 | 14 | 0 | 0 | 0 |
| 35 | Møller et al. 2005 | 183 | 180 | 0 | 0 | 0 | 51 | 99 | 0 | 0 | 0 | 20 | 24 | 0 | 0 | 0 | 110 | 47 | 0 | 0 | 0 |
| 36 | Wong et al. 2003 | 77 | 73 | 0 | 0 | 0 | 12 | 38 | 0 | 0 | 0 | 3 | 2 | 0 | 0 | 0 | 8 | 9 | 0 | 0 | 0 |
| 37 | Pogarell et al. 2002 | 40 | 44 | 0 | 0 | 0 | 5 | 25 | 0 | 0 | 0 | 2 | 2 | 0 | 0 | 0 | 2 | 0 | 0 | 0 | 0 |
| 38 | Pinter et al. 1999 | 44 | 34 | 0 | 0 | 0 | 6 | 14 | 0 | 0 | 0 | 5 | 3 | 0 | 0 | 0 | 6 | 4 | 0 | 0 | 0 |
| 39 | Wermuth et al. 1998 | 33 | 36 | 0 | 0 | 0 | 9 | 20 | 0 | 0 | 0 | 1 | 2 | 0 | 0 | 0 | 5 | 6 | 0 | 0 | 0 |
| 40 | Guttmann et al. 1997 | 83 | 79 | 0 | 0 | 0 | 5 | 36 | 0 | 0 | 0 | 0 | 0 | 0 | 0 | 0 | 33 | 16 | 0 | 0 | 0 |
| 41 | Lieberman et al. 1997 | 179 | 181 | 0 | 0 | 0 | 54 | 108 | 0 | 0 | 0 | 18 | 21 | 0 | 0 | 0 | 39 | 30 | 0 | 0 | 0 |
| 42 | Mohlo et al. 1995 | 12 | 12 | 0 | 0 | 0 | 4 | 5 | 0 | 0 | 0 | 0 | 0 | 0 | 0 | 0 | 0 | 0 | 0 | 0 | 0 |
| 43 | Barone et al. 2015 | 65 | 58 | 0 | 0 | 0 | NA | NA | 0 | 0 | 0 | 1 | 2 | 0 | 0 | 0 | 8 | 9 | 0 | 0 | 0 |

(C: control arm, T1-4: treatment arm 1-4)

**Appendix S10**

**The PRISMA checklist**

| **Section/topic** | **#** | **Checklist item** | **Reported on page #** |
| --- | --- | --- | --- |
| **TITLE** | | |  |
| Title | 1 | Identify the report as a systematic review, meta-analysis, or both. | 1 |
| **ABSTRACT** | | |  |
| Structured summary | 2 | Provide a structured summary including, as applicable: background; objectives; data sources; study eligibility criteria, participants, and interventions; study appraisal and synthesis methods; results; limitations; conclusions and implications of key findings; systematic review registration number. | 2 |
| **INTRODUCTION** | | |  |
| Rationale | 3 | Describe the rationale for the review in the context of what is already known. | 3-4 |
| Objectives | 4 | Provide an explicit statement of questions being addressed with reference to participants, interventions, comparisons, outcomes, and study design (PICOS). | 3-4 |
| **METHODS** | | |  |
| Protocol and registration | 5 | Indicate if a review protocol exists, if and where it can be accessed (e.g., Web address), and, if available, provide registration information including registration number. | A review protocol exists. The protocol has been attached in Appendix S2. |
| Eligibility criteria | 6 | Specify study characteristics (e.g., PICOS, length of follow-up) and report characteristics (e.g., years considered, language, publication status) used as criteria for eligibility, giving rationale. | 5-6  Appendix S2 |
| Information sources | 7 | Describe all information sources (e.g., databases with dates of coverage, contact with study authors to identify additional studies) in the search and date last searched. | 4-5 |
| Search | 8 | Present full electronic search strategy for at least one database, including any limits used, such that it could be repeated. | Appendix S1 |
| Study selection | 9 | State the process for selecting studies (i.e., screening, eligibility, included in systematic review, and, if applicable, included in the meta-analysis). | 5-6 |
| Data collection process | 10 | Describe method of data extraction from reports (e.g., piloted forms, independently, in duplicate) and any processes for obtaining and confirming data from investigators. | 5-6 |
| Data items | 11 | List and define all variables for which data were sought (e.g., PICOS, funding sources) and any assumptions and simplifications made. | 5-6  Appendix S2 |
| Risk of bias in individual studies | 12 | Describe methods used for assessing risk of bias of individual studies (including specification of whether this was done at the study or outcome level), and how this information is to be used in any data synthesis. | 5+  Appendix S3 |
| Summary measures | 13 | State the principal summary measures (e.g., risk ratio, difference in means). | 7 |
| Synthesis of results | 14 | Describe the methods of handling data and combining results of studies, if done, including measures of consistency (e.g., I^2^) for each meta-analysis. | 6-7  +Appendix S5 |

| **Section/topic** | **#** | **Checklist item** | **Reported on page #** |
| --- | --- | --- | --- |
| Risk of bias across studies | 15 | Specify any assessment of risk of bias that may affect the cumulative evidence (e.g., publication bias, selective reporting within studies). | 5-6 |
| Additional analyses | 16 | Describe methods of additional analyses (e.g., sensitivity or subgroup analyses, meta-regression), if done, indicating which were pre-specified. | 7 |
| **RESULTS** | | |  |
| Study selection | 17 | Give numbers of studies screened, assessed for eligibility, and included in the review, with reasons for exclusions at each stage, ideally with a flow diagram. | Fig 1 + Appendix S6 |
| Study characteristics | 18 | For each study, present characteristics for which data were extracted (e.g., study size, PICOS, follow-up period) and provide the citations. | 7 + Appendix S7-9 |
| Risk of bias within studies | 19 | Present data on risk of bias of each study and, if available, any outcome level assessment (see item 12). | 7 +  Appendix S3 |
| Results of individual studies | 20 | For all outcomes considered (benefits or harms), present, for each study: (a) simple summary data for each intervention group (b) effect estimates and confidence intervals, ideally with a forest plot. | 7-9  Fig 3-4 |
| Synthesis of results | 21 | Present results of each meta-analysis done, including confidence intervals and measures of consistency. | 7-9  Table 1 |
| Risk of bias across studies | 22 | Present results of any assessment of risk of bias across studies (see Item 15). | Appendix S3 |
| Additional analysis | 23 | Give results of additional analyses, if done (e.g., sensitivity or subgroup analyses, meta-regression [see Item 16]). | Table 2 |
| **DISCUSSION** | | |  |
| Summary of evidence | 24 | Summarize the main findings including the strength of evidence for each main outcome; consider their relevance to key groups (e.g., healthcare providers, users, and policy makers). | 9-11 |
| Limitations | 25 | Discuss limitations at study and outcome level (e.g., risk of bias), and at review-level (e.g., incomplete retrieval of identified research, reporting bias). | 9-11 |
| Conclusions | 26 | Provide a general interpretation of the results in the context of other evidence, and implications for future research. | 11 |
| **FUNDING** | | |  |
| Funding | 27 | Describe sources of funding for the systematic review and other support (e.g., supply of data); role of funders for the systematic review. | 12 |

*From:*  Moher D, Liberati A, Tetzlaff J, Altman DG, The PRISMA Group (2009). Preferred Reporting Items for Systematic Reviews and Meta-Analyses: The PRISMA Statement. PLoS Med 6(7): e1000097. doi:10.1371/journal.pmed1000097

For more information, visit: **www.prisma-statement.org**.
